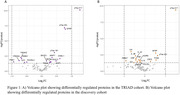# Plasma proteomic profiling of mild cognitive impairment in two cohorts using the NULISAseq CNS Disease panel

**DOI:** 10.1002/alz.092364

**Published:** 2025-01-09

**Authors:** Guglielmo Di Molfetta, Andrea L. Benedet, Bingqing Zhang, Nesrine Rahmouni, Stijn Servaes, Jenna Stevenson, Ilaria Pola, Kaj Blennow, Henrik Zetterberg, Pedro Rosa‐Neto, Nicholas J. Ashton

**Affiliations:** ^1^ Department of Psychiatry and Neurochemistry, Institute of Neuroscience and Physiology, The Sahlgrenska Academy, University of Gothenburg, Mölndal Sweden; ^2^ Department of Psychiatry and Neurochemistry, Institute of Neuroscience and Physiology, The Sahlgrenska Academy, University of Gothenburg, Mölndal, Gothenburg Sweden; ^3^ Alamar Biosciences, Fremont, CA USA; ^4^ McGill University Research Centre for Studies in Aging, Montreal, QC Canada; ^5^ Translational Neuroimaging Laboratory, The McGill University Research Centre for Studies in Aging, Montréal, QC Canada; ^6^ Institute of Neuroscience and Physiology, Department of Psychiatry and Neurochemistry, The Sahlgrenska Academy at University of Gothenburg, Mölndal, ‐ Sweden; ^7^ Paris Brain Institute, ICM, Pitié‐Salpêtrière Hospital, Sorbonne University, Paris France; ^8^ Clinical Neurochemistry Laboratory, Sahlgrenska University Hospital, Mölndal Sweden; ^9^ Department of Neurodegenerative Disease, UCL Institute of Neurology, London United Kingdom; ^10^ Wisconsin Alzheimer’s Disease Research Center, University of Wisconsin School of Medicine and Public Health, Madison, WI USA; ^11^ Hong Kong Center for Neurodegenerative Diseases, Clear Water Bay Hong Kong; ^12^ UK Dementia Research Institute at UCL, London United Kingdom; ^13^ Department of Neurology and Neurosurgery, McGill University, Montréal, QC Canada; ^14^ Centre for Age‐Related Medicine, Stavanger University Hospital, Stavanger Norway; ^15^ Department of Old Age Psychiatry, Institute of Psychiatry, Psychology, and Neuroscience, King’s College London, London, London United Kingdom; ^16^ NIHR Biomedical Research Centre for Mental Health & Biomedical Research Unit for Dementia at South London & Maudsley NHS Foundation, London United Kingdom; ^17^ Wallenberg Centre for Molecular and Translational Medicine, University of Gothenburg, Gothenburg Sweden

## Abstract

**Background:**

Recently, the development of ultra‐sensitive immunoassays has allowed for the detection, in blood, of proteins related to the pathophysiology of Alzheimer’s disease (AD), with phosphorylated tau (p‐tau) being the most promising. However, current methods are often limited by their ability to measure one analyte, lacking the potential for discovery and inclusion of additional biomarkers with supplemental value. In this pilot study, we explored proteomic changes using the novel NUcleic acid Linked Immuno‐Sandwich Assay (NULISA™) platform, focusing on patients with mild cognitive impairment (MCI).

**Method:**

In this study, MCI patients with a confirmed Aβ status were recruited from two independent cohorts. A discovery cohort (mean[SD] age, 73.5 [5.5] years; 25 females [62.5%]) was classified by cerebrospinal fluid Aβ42/40 (Aβ+ =28; Aβ‐ =12). For the second cohort (mean[SD] age, 70.7 [7.31] years; 39 females[42.9%]; CDR 0.5), from the TRIAD study, amyloid positron emission tomography was utilized instead (Aβ+ =47; Aβ‐ =44). We performed the NULISAseq CNS Disease Panel measurements on the plasma samples (n=131) in two separate batch analyses. LIMMA models were used to evaluate differential expression of protein NPQ values between the two MCI (Aβ+ Vs. Aβ‐) patient groups, with a total of 121 proteins included in the analysis.

**Result:**

In the discovery cohort (n=40), only p‐tau217 survived multiple comparison (Log_2_FC, 1.49; P^adj^ <0.001). Further targets were present with a significant unadjusted p‐value (P <0.05) (Figure 1b). In the larger TRIAD cohort (n=91), p‐tau217 (Log_2_FC, 1.29; P^adj^ <0.001), p‐tau231 (Log_2_FC, 0.76; P^adj^ <0.05), p‐tau181 (Log_2_FC, 0.42; P^adj^ <0.05) and GFAP (Log_2_FC, 0.78; P^adj^ <0.05) remained significant after adjusting for multiple testing. Similarly to the discovery cohort, additional targets were identified as significantly changed with an unadjusted p‐value (P<0.05) (Figure 1A). This included PARK7, which was the only target present in both.

**Conclusion:**

In this study, consisting of two independent groups of MCI patients characterized by Aβ burden, we utilized the NULISAseq CNS Disease Panel to identify dysregulated proteins of the prodromal stage of AD. This novel multiplex technology continues to demonstrate the importance of p‐tau217, p‐tau231, p‐tau181 and GFAP as indicators of cerebral amyloid deposition while offering additional markers that may increase their utility.